# Social jet lag is associated with core symptoms in 2-3-year-old children with autism spectrum disorders

**DOI:** 10.3389/fpsyt.2025.1574814

**Published:** 2025-05-16

**Authors:** Hongyu Chen, Ting Yang, Jie Chen, Yuan Ding, Xueli Xiang, Qiuhong Wei, Qiuhong Mou, Binlin Yuan, Binyue Hu, Danyang Zhang, Dan Ai, Tingyu Li

**Affiliations:** ^1^ Children’s Nutrition Research Center, Children’s Hospital of Chongqing Medical University, Chongqing Key Laboratory of Child Neurodevelopment and Cognitive Disorders, Ministry of Education Key Laboratory of Child Development and Disorders, National Clinical Research Center for Child Health and Disorders, Chongqing, China; ^2^ Growth, Development, and Mental Health of Children and Adolescence Center, Children’s Hospital of Chongqing Medical University, Chongqing, China

**Keywords:** autism spectrum disorder, social jet lag, sleep patterns, core symptoms, circadian rhythm

## Abstract

**Background:**

Social jet lag (SJL) is a form of circadian rhythm misalignment caused by the mismatch between social schedules and biological clocks, which is associated with cognition, behavior, and emotion in children. However, social jet lag among children with autism spectrum disorders (ASD) and its impacts are unknown.

**Methods:**

This cross-sectional study recruited 2-7-year-old children with ASD from special education institutions and outpatient clinics. The Children’s Sleep Habits Questionnaire (CSHQ) assessed children’s sleep. SJL was calculated as |weekend sleep midpoint - weekday sleep midpoint|. Sleep adequacy was determined based on the National Sleep Foundation’s recommendations. Core symptoms were evaluated using the Childhood Autism Rating Scale (CARS), Social Responsiveness Scale (SRS), and Autism Behavior Checklist (ABC). Developmental level was assessed using the Gesell Developmental Scale.

**Results:**

1) The prevalence of sleep problems was 49.8% and the mean CSHQ total score was 48.04 in ASD. There are significant differences in sleep patterns between weekends and weekdays, characterized by later bedtimes, delayed wake-up times, increased total sleep duration, and reduced prevalence of sleep deficiency during weekends. 2) The 2-3-year-old group had the highest rates of sleep insufficiency (80.77% on weekdays; 82.17% on weekends). There were no significant differences in sleep duration across different age groups, with the median sleep duration ranging from 9.5 to 10 hours. 3) Median SJL in each age group was 0.25 h (2–3 years), 0.5 h (3–4 years), 0.42 h (4–5 years), and 0.5 h (≥5 years), respectively. In children aged 2–3 years, SJL was significantly positively correlated with core symptoms 4) SJL was observed to be weakly associated with developmental level of personal-social only in the ≥ 3-year-old group (*r* = 0.100, *P* = 0.042).

**Conclusion:**

Our study found for the first time a correlation between SJL and core symptoms in 2-3-year-old children with ASD. This finding suggests that SJL may have a potentially negative impact on core symptoms in ASD. Therefore, it is crucial to emphasize the importance of regular routines for ASD, especially in younger children.

## 1 Introduction

Autism Spectrum Disorder (ASD) is a neurodevelopmental condition characterized by social communication deficits, restricted interests, and repetitive behaviors (1). Sleep disturbances, including delayed sleep onset, frequent nighttime awakenings, and circadian rhythm disruptions, are prevalent in ASD (2). There are two main mechanisms for sleep regulation: the biological clock (also known as circadian rhythm) and sleep homeostasis (3). Biological clock is located in the suprachiasmatic nucleus (SCN) of hypothalamus, which is the master clock that regulates life’s activities (4). Internal clock has to be synchronized with the rhythms of the external environment, and this is accomplished through light (5). There are social activities in human society, including work, study, etc., so individuals also need synchronize the internal biological clock with social activities. When they are not synchronized, social jet lag (SJL) occurs (6).

Social jet lag is an easily overlooked manifestation of circadian rhythm disorders, usually reflected in differences in sleep patterns between weekdays and weekends (SJL = |weekend sleep midpoint - weekday sleep midpoint|) (6). SJL affects mental and physical health, including metabolism, cardiovascular health, sleep, anxiety and depression (7–14). However, most of the relevant studies have been conducted in adults and adolescents, with fewer in children, especially in ASD. A large multicenter survey of preschool children in China showed that longer SJL was positively associated with children’s emotional and behavioral problems (15). In children with attention deficit and hyperactivity disorder (ADHD), SJL was positively correlated with their cognitive abilities and core symptoms (16). In ASD, a study that included 171 individuals aged 5–21 years investigated the correlation of SJL with age and sleep, but no further reports of SJL with core symptoms (17). Autistic children may experience more severe SJL-related consequences due to their inherent clinical characteristics, such as social impairments and difficulties in adapting to the environment. Currently, there is a limited number of reports on social jet lag in children with ASD, and the associations with the core symptoms have not yet been clarified, so related studies are needed.

This study has the following main objectives: 1) investigate the sleep patterns of children with ASD during weekdays and weekends, 2) examine the sleep patterns and the characteristics of social jet lag in children with ASD of different ages, 3) explore the correlation between social jet lag and the core symptoms, and developmental level of ASD. We hope that our study will enable more researchers to understand the characteristics the possible negative effects of social jet lag in children with ASD, and provide parents and clinicians with practical guidance suggestions to promote the establishment of a regular routine.

## 2 Methods

### 2.1 Participants

This cross-sectional study enrolled autistic children from Chongqing, China. Inclusion criteria: 1) 2–7 years old; 2) DSM-5-confirmed ASD diagnosis by developmental -behavioral pediatricians; 3) informed consent from caregivers. Exclusion criteria: 1) suffering from other serious diseases; 2) non-completion of questionnaires.

The study was approved by the Ethics Committee of Children’s Hospital of Chongqing Medical University (No. 121-1/2018) and registered at the Chinese Clinical Trial Registry (ChiCTR2000031194).

### 2.2 Clinical assessments

#### 2.2.1 Sleep conditions

The Children’s Sleep Habits Questionnaire (CSHQ) was used to assess children’s sleep and consisted of 33 scored entries as well as a survey of bedtime and wake up time (18). Recent studies have suggested that using the original score of 41 to define sleep problems may be overestimated and that using a score of 48 may be more reasonable (19–21), so we used this as the cutoff in subsequent analyses. Sleep patterns were obtained from parents answering the following questions: bedtime and wake up time on weekdays/weekends. Sleep duration is the interval between bedtime and wake-up time. Sleep midpoint = wake up time - (sleep duration/2). SJL = |weekend sleep midpoint − weekday sleep midpoint| (22). Adequacy of sleep is determined based on the American Sleep Foundation’s recommended hours of sleep, which are 11–14 hours for toddlers, 10 hours for ages 3-6, and 9 hours for ages 6-14 (23).

#### 2.2.2 Core symptoms

Core symptoms in children with ASD were assessed using the Childhood Autism Rating Scale (CARS), Social Responsiveness Scale (SRS), and Autism Behavior Checklist (ABC). The CARS is a 15-item scale that assesses the severity of autism (24). The SRS contains 65 items assessing the dimensions of social awareness, social cognition, social communication, social motivation, and autistic mannerisms, with a total score range of 0-195 (25). The ABC scale contains 57 items assessing 5 dimensions: sensory, relating, body and object use, language, and social and self-help, with a total score range of 0-158 (26).

#### 2.2.3 Developmental level

The developmental level was assessed using the Gesell Developmental Scale (GDS), consisting of adaptive behavior, gross motor, fine motor, language, and personal-social (27). Total developmental quotient (DQ) is the mean of the five dimensions.

### 2.3 Statistical analysis

Data were analyzed using SPSS 25.0. Continuous variables were expressed as mean ± standard deviation (M ± SD) or Median (25th percentile to 75th percentile) [M (P25-P75)], group comparisons using one-way Analysis of Variance (ANOVA) or Kruskal-Wallis tests. Categorical variables were expressed as frequencies (percentages) [N (%)], group comparisons using chi-square tests. Paired t-tests, Wilcoxon signed-rank tests, and McNemar’s tests were employed for intra-group comparisons, with selection criteria based on data characteristics. Bonferroni correction was applied for multiple comparisons. Correlation analyses were performed based on data distribution. Pearson’s correlation coefficient was applied to variables meeting normality assumptions, while Spearman’s rank-order correlation was employed for non-normally distributed variables. Statistical significance was set at *P* < 0.05.

## 3 Results

### 3.1 Study population

A total of 701 children with ASD were included in this study, and 36 children were excluded because their questionnaires contained missing items (e.g., filled in the time of going to sleep, missing the time of waking up; filled in the sleep situation on weekdays, missing the weekend). Finally, 545 boys and 121 girls were included in this study, with an age distribution of 3.82 (3.13,4.61) years. The prevalence of sleep problems was 49.8% and the mean CSHQ total score was 48.04.

### 3.2 Sleep patterns during weekdays and weekends in children with ASD

As shown in **Table 1**, there were significant differences in the sleep patterns of children with ASD between weekdays and weekends. More children slept later, got up later, slept more hours, and had lower rates of sleep insufficiency on weekends than on weekdays (*P* < 0.05), suggesting that weekend routines may have compensated for weekday sleep insufficiency. Both the World Sleep Association and the National Health Commission of China recommend that children should go to bed before 21:00. In this survey, the percentage of autistic children went to bed after 21:00 on weekdays was 87.22%, which further increased to 92.48% on weekends (*P* < 0.05).

**Table 1:** Comparison of sleep patterns on weekdays and weekends in children with ASD, n=665.

Variables	Weekdays	Weekends	*χ^2^/Z*	*p*	*r*/*φ*
Sleeping time	22:00(21:30-22:30)	22:03(21:45-23:00)	-14.772	<0.001	0.573
Waking time	07:30(07:00-08:00)	08:00(07:30-08:50)	-15.353	<0.001	0.595
Sleep duration	9.67(9.00-10.00)	10(9.33-10.5)	-8.672	<0.001	0.336
Sleep insufficiency	395(59.40)	327(49.17)	21.811	<0.001	0.181
Go to sleep after 21:00	580(87.22)	615(92.48)	32.529	<0.001	0.221

### 3.3 Comparison of sleep patterns and social jet lag in children with ASD in different age groups

As shown in **Table 2**, children in the ≥5 years group slept the earliest during weekdays and weekends compared to the other age groups, [21:30 (21:00, 22:07), *P* < 0.05] on weekdays and [22:00 (21:30, 22:30), *P* < 0.05] on weekends, but still 81.5% and 90.7% of them went to sleep after 21:00. It can be seen that the children with ASD had a higher incidence of late sleep in this investigation.

**Table 2:** Comparison of sleep patterns and social jet lag in children with ASD at different age groups.

Variables		2-3y (130)	3-4y (240)	4-5y (187)	≥5y (108)	*H/χ2*	*P*
Sleeping time	weekdays	22:00 (21:30,22:30)^a^	22:00 (21:30,22:30)^a^	22:00 (21:20,22:20)^ab^	21:30 (21:00,22:07)^b^	13.957	0.003
	weekends	22:20 (21:50,22:36)^ab^	22:23 (22:00,23:00)^a^	22:00 (21:40,23:00)^ab^	22:00 (21:30,22:30)^b^	13.262	0.004
Waking time	weekdays	07:30 (07:00,08:30)^a^	07:30 (07:08,08:00)^a^	07:30 (07:00,08:00)^ab^	07:30 (07:00,08:00)^b^	12.664	0.005
	weekends	08:00 (07:30,09:00)	08:06 (07:30,09:00)	08:00 (07:30,08:30)	08:00 (07:22,08:45)	6.483	0.090
Sleep duration	weekdays	9.67 (9,10.38)	9.50 (9,10.07)	9.83 (9,10.17)	9.67 (9,10)	3.782	0.286
	weekends	10 (9.48,10.52)	10 (9,10.5)	10 (9.48,10.5)	10 (9.5,10.5)	0.363	0.948
Sleep insufficiency	weekdays	105 (80.77)^a^	139 (57.92)^b^	103 (55.08)^b^	48 (44.44)^b^	36.297	<0.001
	weekends	106 (82.17)^a^	107 (44.58)^b^	80 (42.78)^b^	34 (31.48)^b^	71.884	<0.001
Go to sleep after 21:00	weekdays	114 (87.69)^ab^	221 (92.08)^b^	156 (83.42)^a^	88 (81.48)^a^	10.607	0.014
	weekends	120 (92.31)	227 (94.58)	169 (90.37)	98 (90.74)	3.116	0.374
Social jet lag		0.25 (0,0.5)^a^	0.5 (0.08,0.75)^b^	0.42 (0.01,0.75)^ab^	0.5 (0.08,0.75)^b^	15.06	0.002

Different letters indicate significant differences, *P* < 0.05, and the same letters indicate non-significant differences, *P* > 0.05.

Sleep insufficiency tended to decrease in older age groups, and was significantly higher in the 2-3-year-old group (*P* < 0.05), 80.77% on weekdays and 82.17% on weekends, compared to 44.44%-57.92% (weekdays) and 31.48%-44.58% (weekends) in other age groups. There was no difference in sleep duration between the groups, but the fact is that the younger the age, the more sleep is needed. According to the recommendations of the American Sleep Foundation, the recommended hours of sleep for children aged 2–3 years should be more than 11 hours, but here the P75 for sleep duration is 10.38 hours on weekdays and 10.52 hours on weekends.

The median of SJL was 0.5 h, 0.42 h, and 0.5 h for the 3-4, 4-5, and ≥5 year old groups, respectively, which were not significantly different from each other. The 2-3-year-old group had the shortest SJL of 0.25 (0, 0.5) h, which was significantly lower than that in 3–4 and ≥5 year old age groups (*P* < 0.05).

### 3.4 Correlations between social jet lag and core symptoms in children with ASD in different age groups

Since the social jet lag among the 3-4, 4-5, and ≥5-year-old groups was similar and showed no significant differences, we merged them into a single group. The association between SJL and core symptoms was assessed using Spearman’s rank-order correlation analysis. As shown in **Table 3** below, in the 2-3-year-old group, SJL was positively correlated with the following scores: ABC relating (*r* = 0.178, *P* = 0.050), ABC language (*r* = 0.297, *P* = 0.001), ABC total score (*r* = 0.178, *P* = 0.050), SRS communication (*r* = 0.163, *P* = 0.065), SRS motivation (*r* = 0.181, *P* = 0.039), SRS autistic mannerisms (*r* = 0.215, *P* = 0.014), and SRS total score (*r* = 0.208, *P* = 0.018). In the ≥3 year old group, no correlation was found between SJL and core symptom scores.

**Table 3:** Correlation between social jet lag and core symptoms in children with ASD at different age groups.

Variables	2-3y (130)		≥3y (535)	
	*r*	*P*	*r*	*P*
ABC				
Sensory	0.077	0.397	0.028	0.534
Relating	0.178	0.050	0.061	0.179
Body and object use	0.061	0.502	-0.040	0.375
Language	0.297	0.001	0.026	0.562
Social and self-help	0.103	0.259	0.042	0.358
Total score	0.178	0.050	0.023	0.603
SRS				
Social awareness	0.126	0.154	0.036	0.411
Social cognition	0.142	0.107	0.084	0.052
Social communication	0.163	0.065	0.052	0.235
Social motivation	0.181	0.039	0.045	0.296
Autistic mannerisms	0.215	0.014	0.031	0.476
Total score	0.208	0.018	0.057	0.187
CARS	0.095	0.317	-0.022	0.627

### 3.5 Correlations between social jet lag and developmental level in children with ASD in different age groups

The association between SJL and developmental level was assessed using Spearman’s rank-order correlation analysis. In **Table 4**, the ≥ 3-year-old group, the correlation between developmental level of personal-social and SJL was extremely weak (*r* = 0.100, *P* = 0.042) and can be considered irrelevant. Overall, no correlation between developmental level and social jet lag was found.

**Table 4:** Correlation between social jet lag and developmental level in children with ASD at different age groups.

Variables	2-3y (130)		≥3y (535)	
	*r*	*P*	*r*	*P*
Adaptive behavior	-0.024	0.810	0.045	0.357
Gross motor	-0.077	0.436	0.075	0.126
Fine motor	-0.051	0.609	0.041	0.411
Language	-0.096	0.332	0.078	0.114
Personal-social	-0.161	0.102	0.100	0.042
DQ	-0.102	0.301	0.076	0.124

## 4 Discussion

In this study, the median of bedtime for children with ASD by age ranged from 21:30 to 22:23, waking up time from 7:30 to 8:06, and sleep duration from 9.5 to 10 hours. This is generally consistent with previous reports. Zou’s (28) study showed that autistic children under 6 years old went to sleep at 22:30 on weekdays and 23:00 on weekends, woke up at 7:30 and 8:00, respectively, and slept for about 10 h. Among children aged 3–5 years with ASD, Li’s (29) survey showed that their average sleep duration on weekdays and weekends was 9.74 h and 9.99 h, respectively, and Iwamoto (30) reported a bedtime of 21:45 and a wake-up time of 7:25, with an average sleep duration of 8.85 h. In Anders’ (31) study, children with ASD aged 2-5.5 years went to bed at 21:00 and slept for approximately 10 h. Due to variations in sample sizes, survey instruments used, and cultural regions, the results of the surveys varied slightly.

Our study indicates that children with ASD exhibit significant differences in sleep patterns between weekends and weekdays, characterized by later bedtimes, delayed wake-up times, increased total sleep duration, and reduced prevalence of sleep deficiency during weekends. This may be associated with more relaxed schedules on weekends. Parents often grant children greater autonomy in time management, resulting in delayed sleep timing. However, while such compensatory sleep behavior partially alleviates weekday sleep deprivation, it may also induce circadian rhythm disruption and other adverse effects, potentially compromising overall sleep quality (32, 33). Several domestic and international guidelines recommend that children should be in bed before 21:00 (34, 35). However, our survey reveals that 81.48%-92.08% of ASD children across different age groups go to bed after 21:00 on weekdays, with this proportion further increasing to 90.37%-94.58% on weekends. These findings demonstrate that delayed bedtime is prevalent among ASD children of all ages. Future interventions should emphasize cultivating regular sleep-wake routines, including promoting early bedtimes, trying to follow consistent bedtime and wake-up times on weekdays and weekends, and so on.

In different age groups exhibit distinct characteristics in terms of sleep patterns and social jet lag, especially in the 2-3-year-old group. The prevalence of insufficient sleep is most severe in the 2-3-year-old group (80%), whereas it ranges from 31% to 58% in the other three older age groups. According to the recommendations of the National Sleep Foundation (34), children aged 2–3 years should have a sleep duration of more than 11 hours. However, the results of this survey show that the P75 sleep duration is 10.38 hours on weekdays and 10.52 hours on weekends. In addition, although the SJL in the 2-3-year-old group is the shortest compared with other age groups, its negative impact on core symptoms is the greatest, especially in terms of language and communication, indicating that younger ASD children are more sensitive to changes in daily routines. This may be related to the physiological and psychological developmental characteristics of this age group. Nervous system develops rapidly before the age of 3 (36). The Lancet has also emphasized the importance of development before the age of 3 in its series of articles on early child development (37–39). The period from conception to 3 years old is the time when children are most vulnerable to adverse exposures and when interventions are most beneficial. The latest Guidelines on Early Infant and Toddler Development Services issued by our Health Commission 2025 expresses a similar viewpoint, clearly stating that the period before the age of 3 is a critical period for children’s growth and development.

This is the first time we have reported the association between SJL and core symptoms in children with ASD aged 2–7 years. In a study involving 171 children with ASD aged 5–21 years, the overall SJL was 0.72 hours and was positively correlated with age, but the authors did not explore the relationship between social jet lag and core symptoms (17). Compared to this study, we had a larger sample size and investigated SJL in different age subgroups. In addition, we investigated the association between SJL and core symptoms and developmental levels in ASD, revealing its potential significance in this population. In a multicenter survey in China (40), longer SJL was associated with poorer psychological health in children and adolescents. Another study in preschool showed SJL was positively correlated with overall emotional and behavioral problems (15). In ADHD, SJL is considered an important predictor of ADHD symptoms (41). These studies reveal the important role of SJL in children’s emotion, behavior and so on from different populations, and indirectly support our research conclusions.

In the group of autistic children aged ≥3 years, the SJL is higher than that in the 2-3-year-old group. However, no correlation between SJL and core symptoms has been found yet. This may be related to the fact that more children with ASD receive behavioral interventions or enter kindergarten after the age of 3 (42). On one hand, fixed intervention programs and kindergarten routines can help children with ASD establish regular daily routines on weekdays. However, the more relaxed family environment on weekends leads to an increase in SJL. On the other hand, behavioral interventions may to some extent mitigate the negative impact of SJL on core symptoms. In addition, as the circadian rhythm regulation ability of older children is enhanced, their resistance to external disturbances may also be improved. However, this does not mean that sleep problems and circadian rhythm disorders in older children can be ignored.

Another noteworthy point is that the majority of autistic children in all age groups go to bed after 21:00, but in ≥5-year-old group the bedtime is the earliest. This indicates that late bedtime is not only caused by irregular daily routines, but may also be related to some physiological characteristics of ASD, involving deeper-level mechanism discussions, such as melatonin. It requires further research.

Based on the results of this study, we propose the following recommendations: 1) Families, institutions, and kindergartens should enhance communication to cultivate regular daily routines in children with ASD. 2) Children’s daily routines are closely related to parental sleep habits and parenting styles. Therefore, parents should be aware of their important role in shaping children’s sleep habits. On the one hand, parents should set a positive example for their children by adjusting their own sleep behavior; on the other hand, they need to create a good sleep environment for their children, set a reasonable sleep schedule and supervise the implementation. 3) For children with ASD aged 2–3 years, special attention should be paid to inconsistencies between weekday and weekend routines to alleviate the negative impacts of irregular sleep patterns.

Several limitations of this study should be noted. First, subjective questionnaire was used to survey the bedtime and wake-up time. In future studies, we plan to incorporate actigraphy to objectively assess children’s sleep patterns and expand data collection to include parental sleep profiles. Moreover, this study only preliminarily explored the social jet lag in children with ASD and its association with core symptoms. Further research is needed to comprehensively investigate the role of SJL in autism from multiple dimensions. For instance, functional magnetic resonance imaging (fMRI) could be employed to characterize the relationship between SJL and functional connectivity within social brain networks, as well as to explore the interaction between SJL and genes in children with ASD.

## 5 Conclusion

Our study found for the first time a correlation between SJL and core symptoms in 2-3-year-old children with ASD. This finding suggests that SJL may have a potentially negative impact on core symptoms in ASD. Therefore, it is crucial to emphasize the importance of regular routines for ASD, especially in younger children.

## Data Availability

The raw data supporting the conclusions of this article will be made available by the authors, without undue reservation.
