# Supplementary material for: Social jet lag is associated with core symptoms in 2-3-year-old children with autism spectrum disorders
Source: Front Psychiatry. 2025 May 16;16:1574814. doi: 10.3389/fpsyt.2025.1574814 (PMC12122480; doi:10.3389/fpsyt.2025.1574814)
Supplement: Supplementary file 1 [file Table1.docx]

| Supplementary table 1. Correlation between social jet lag and CSHQ scores in children with ASD at different age groups | | | | | |
| --- | --- | --- | --- | --- | --- |
| Variables | 2-3y (130) | |  | ≥3y (535) | |
|  | *r* | *P* |  | *r* | *P* |
| Bedtime resistance | -0.013 | 0.886 |  | 0.094 | 0.029 |
| Sleep onset delay | 0.055 | 0.537 |  | 0.034 | 0.430 |
| Sleep duration | 0.156 | 0.076 |  | -0.009 | 0.838 |
| Sleep anxiety | 0.026 | 0.769 |  | 0.076 | 0.080 |
| Night waking | -0.05 | 0.575 |  | -0.025 | 0.568 |
| Parasomnia | 0.017 | 0.851 |  | 0.085 | 0.050 |
| Sleep-disordered breathing | 0.006 | 0.948 |  | 0.016 | 0.714 |
| Daytime sleepiness | 0.218 | 0.013 |  | 0.185 | ＜0.001 |
| Total score | 0.149 | 0.092 |  | 0.148 | 0.001 |
